# Sampling of Atmospheric Precipitation and Deposits for Analysis of Atmospheric Pollution

**DOI:** 10.1155/JAMMC/2006/26908

**Published:** 2006

**Authors:** K. Skarżyńska, Ż Polkowska, J. Namieśnik

**Affiliations:** 1 Department of Analytical Chemistry Chemical Faculty Gdańsk University of Technology 11/12 Narutowicza Street Gdańsk 80-952 Poland

## Abstract

This paper reviews techniques and equipment for collecting precipitation samples from the atmosphere (fog and cloud water) and from atmospheric deposits (dew, hoarfrost, and rime) that are suitable for the evaluation of atmospheric pollution. It discusses the storage and preparation of samples for analysis and also presents bibliographic information on the concentration ranges of inorganic and organic compounds in the precipitation and atmospheric deposit samples.


Hindawi Publishing Corporation
Journal of Automated Methods and Management in Chemistry
Volume 2006, Article ID 26908, Pages 1–19
DOI 10.1155/JAMMC/2006/26908

### Sampling of Atmospheric Precipitation and Deposits for Analysis of Atmospheric Pollution

**K. Skarżyńska, Ż Polkowska, and J. Namieśnik**

*Department of Analytical Chemistry, Chemical Faculty, Gdańsk University of Technology, 11/12 Narutowicza Street, Gdańsk 80-952, Poland*

Received 15 August 2005; Accepted 31 October 2005

This paper reviews techniques and equipment for collecting precipitation samples from the atmosphere (fog and cloud water) and from atmospheric deposits (dew, hoarfrost, and rime) that are suitable for the evaluation of atmospheric pollution. It discusses the storage and preparation of samples for analysis and also presents bibliographic information on the concentration ranges of inorganic and organic compounds in the precipitation and atmospheric deposit samples.

Copyright © 2006 K. Skarżyńska et al. This is an open access article distributed under the Creative Commons Attribution License, which permits unrestricted use, distribution, and reproduction in any medium, provided the original work is properly cited.

### 1. INTRODUCTION

The tremendous dynamics of the atmosphere makes it the main propagation path for air pollution and its transport between the various elements of the environment in the form of dust, gases, and aerosols. Pollution, depending on its properties and on meteorological conditions, is subject to scattering and transformation during atmospheric transport. Most of the pollutants eventually return to the earth's surface, often at great distances from their sources. Their return may be through precipitation or absorption of gaseous pollutants and aerosols by surface waters, the vegetation cover, or the soil. Of these, wet deposition plays the greatest role in feeding atmospheric pollutants back to the earth's surface in regions distant from their sources.

Atmospheric precipitation is the result of condensation of atmospheric water vapour due to adiabatic cooling. Precipitation falls down to the surface in the form of rain, snow, drizzle, snow pellets, and hail; floats in the air in the form of clouds and fog, and settles on surfaces in the form of dew, hoarfrost, and rime (see Figure 1).

Environmental protection begins with identification and definition of the kind and degree of pollution and its analysis and monitoring. During the past 20 years the field of analysis and monitoring of atmospheric deposition has grown dramatically because we now recognize that the quantity and quality of transported pollutants, as well as the range of their interactions, can be a good indicator of the degree of atmospheric pollution in a given geographical region. An extensive review of the design and basic parameters of samplers for rain precipitation and runoff waters appeared in 2002 [1]. This paper is prompted by the growing interest in the sampling of other atmospheric deposits, particularly fog and clouds, dew, hoarfrost and rime, that began around 1990. The basic characteristics of the forms of precipitation that will be discussed in this paper are presented in Table 1.

### 2. IMPORTANCE OF REPRESENTATIVE SAMPLES

To be a source of reliable analytical information a sample must be representative of the object or distribution from which it is extracted. In practice the samples we analyze can only be a small fragment of the objects of interest. Hence, we must take care to insure that the samples are as representative as possible [2]. Sampling has a special significance for all analytical processes; in a sense, it is the critical point of analysis. Errors committed at this stage cannot be estimated nor can their effect upon the result of the analysis be reduced.

One of the most important ways in which sampling can fail to be representative is through sample contamination. For sampling of atmospheric deposits, the proper preparation of the sampler is an important factor for minimizing sample contamination. The most obvious step is also the most important—cleaning the sampler before collecting a sample. A standard cleaning procedure includes, among other things, washing in deionized water [3–5] and/or for example, rinsing with acetone [6]. Collecting vessels are also washed in water with detergents [7], in aqueous solutions of nitric acid [7], distilled water, and eventually rinsed with deionized water [8, 9]. Subsequent handling of samples in

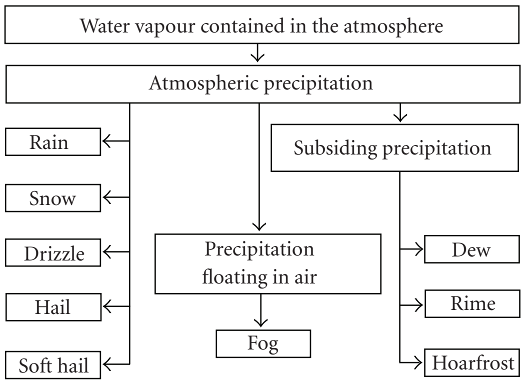

Figure 1: Types of atmospheric precipitation and deposits.

accord with good laboratory practice is, of course, also a requirement for minimization of sample contamination.

We now turn to our review of the designs of samplers for the collection of fog and cloud water, dew, hoarfrost, and rime.

### 3. SAMPLERS FOR THE COLLECTION OF FOG AND CLOUD WATER SAMPLES

As previously noted, transport of anthropogenic pollutants through the atmosphere is an important means of their worldwide distribution. Airborne pollutants can be transferred to aquatic and land environments via mechanisms such as wet and dry deposition and air/water partitioning. Fog has recently attracted the attention of the scientific community as a potentially important deposition mechanism. Organic and inorganic pollutants distribute themselves between the vapour and aqueous phases, of fog and between the particles present in both phases and chemical reactions can occur within fog droplets. When fog droplets subsequently evaporate, the chemicals contained in them can be deposited on surfaces in contact with the fog [10].

Over the last 20 years, investigators have examined the chemical compositions of cloud and fog [7, 11–13]. They have studied the processes occurring in atmospheric particles and developed sample collection methods [14, 15]. Collecting samples of fog and cloud water is often more complicated than collecting samples of precipitation or runoff water [1], since we need to extract the water from the cloud or fog. Many of the methods for fog and cloud water sampling are based on instruments originally designed for quantitative meteorological measurements of the amount of water in clouds or fog. Fog and cloud water samplers that are used for the evaluation of pollutants differ from those used for water measurements, because they must not only perform efficient, quantitative water collection, but must also avoid collection of submicron "nonactivated" aerosols, preserve the size and chemical composition of droplets through all the stages of collection, and provide rapid collection of relatively large amounts of liquid water for wet chemical analysis. As with any instrument, the collector should also be easy to use and automate, and should require minimal maintenance.

Cloud and fog water collectors operate primarily on the principle of inertial impaction on a plane surface, a standard technique also used for the collection of dry aerosol particles. Collection efficiency curves, which show the percentage of particles of any size which are collected as a function of the particle size, indicate that the instruments based on inertial impaction generally provide more reliable results than other methods [16].

The efficiency curves of inertial impactors show a sharp division (cutoff) between the droplets collected and those which are not. Marple and Willeke [17] formulated design criteria for inertial impactors that enable construction of dry aerosol impactors with well-defined cutoff characteristics. Their techniques had to be adapted, however, to the special needs of liquid water sampling [16]. Single stage cloud water impactors, based on the adapted techniques, have been used for several years to perform studies of cloud and fog water chemical composition [16]. They are used in both active collectors, where flow of air containing the droplets is forced by means of a suitable mechanical device, and passive collectors, where natural circulation of air (wind) is utilized. There is a wide variety of passive and active collectors available for various ambient conditions.

### 4. PASSIVE COLLECTORS

Passive collectors are often simpler in operation and may be used in windy environments. However, their impaction characteristics (cutoff) are less controllable. The collecting elements (impaction plane) for passive collectors may vary from flat surface to solid elements like rods; tubes as well as strings, ropes, filaments, screens, and meshes have also been used.

The simplest passive fog sampler is the deposition plate, typically a horizontal plate on which fog droplets are allowed to settle [18]. This sampler may suffer from contamination due to dry deposition and dew formation, which leads to significant biases toward errors with large particle measurements. The design is attractive on its own, but its disadvantages restrict its applications.

The next simplest fog sampler is the string screen sampler in which fog is collected through the impaction of fog droplets on a string screen. After collision the droplets adhere to the string and drop along the strings to a collection tray. String screen samples can be also in an active version.

In a published paper [19], a passive collector is described (Figure 2), consisting of a 2 m tall collection, of two horizontal disks 20 cm in diameter, installed vertically on frames at a distance of 40 cm from each other. Between the disks, Nylon strings of 0.2 mm diameter are stretched into two rows. The fog water collected on the strings is stored in a 500 mL polyethylene bottle. Collection area was 314 $cm^2$. A hood above the collector prevents rain from diluting the fog sample. The sampler was set up in a 1 m high PVC tube to protect it from direct radiation and light.

The principle of the work of the collector described in [20] (Appalachian Mountain Club/Worcester Polytechnic Institute (AMC/WPI)) is based on the utilisation of wind to transfer particles to Teflon collecting strands. The cloud

Table 1: The characteristics of some atmospheric precipitation types.

| Atmospheric precipitation or deposits | Characteristics |
|---|---|
| Cloud | Water droplets of microscopic size (diameters not exceeding 100 $\mu$m) and/or ice crystals floating in the air due to microturbulences counteracting their gravitational sedimentation. |
| Fog | Suspension of very small water droplets with diameters below 0.05 mm or ice crystals in air. It is formed from the result of cooling from the ground layer of air to the dew–point temperature–reaching the state of saturation with water vapour. From the sample collection point of view both cloud and fog present similar challenges. |
| Dew | Deposit of water droplets forming on the surface of ground and objects on earth's surface or close to it due to condensation of water vapour contained in surrounding air. Generally, it is caused by night-time radiation of heat. Dew originates also when warm and humid airflows above a cooled-down substratum, whose temperature is lower than the saturation point of the inflowing air mass. |
| Hoarfrost | Deposit of ice crystals forming generally on horizontal surfaces. The appearance and size of the deposit depend on thermal properties of the substratum and on air humidity. It occurs with distinct differences between air and ground temperatures and the ground surface temperature must be negative but the air temperature can be positive. It is often linked with ground frost. |
| Rime | Granular or crystalline deposit (generally both forms of deposit appear simultaneously) settling most easily on thin fibrous objects windward, at negative air temperature and with fog. The amount of the deposit depends on the density and duration of fog. |

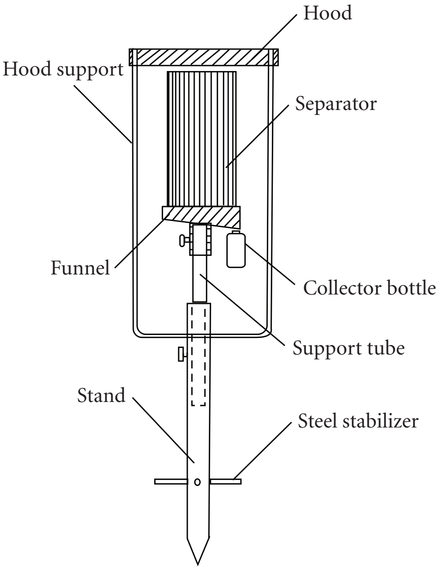

Figure 2: Structure of a passive fog water collector [19].

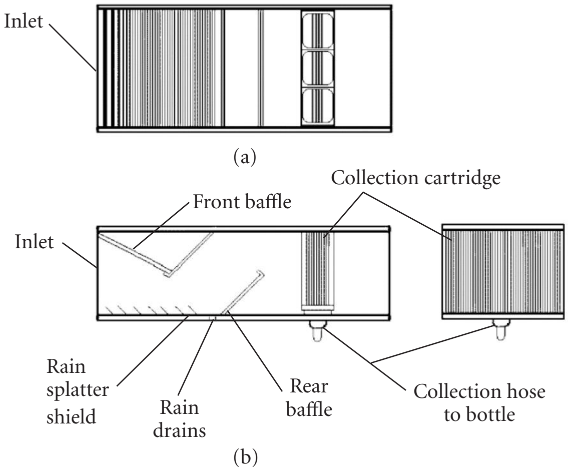

Figure 3: Structure of a passive cloud water collector (AMC/WPI) [20]: (a) top view, (b) side view.

water droplets are collected principally by the mechanism of inertial impaction on Teflon strings. Exclusion of the heavier rain droplets is accomplished by both the placement of the collection strands deep within the collection box and by a baffle system (Figure 3). The airflowing through the baffles is restricted and forced to turn, causing it to accelerate. The rain droplets, with their greater inertia, overcome the viscous drag effects of the airstream and pass out of it, impacting on the lower baffle. A drain below the baffle permits the separated rain water to leave the collector. The cartridge containing the collection strands is located behind the baffle to allow adequate expansion of the airflow and thus to maximize the utilization of the collection surface area. The distance back from the top of the entrance of the collection box to the upper lip of the lower baffle was selected for its theoretical ability to remove free-falling droplets $>200\,\mu$m at winds of 0–10 m/s and droplets $>500\,\mu$m at winds of 0–25 m/s. These values represent the minimum ability of the collector to exclude rain and drizzle. To prevent contamination of the cloud water sample by rain, baffles have lips to prevent the impacted rain water from running to the edge of the baffle and from becoming reentrained in the accelerated airflow. The upper baffle has a small reservoir where water collects prior to draining out holes drilled on the side of the collector. To prevent rain splatter on the lower surface of the collector from reentering the airstream, a series of vanes set at 40 degrees is positioned in front of the lower baffle. Rain water collected by these vanes leaves the collector at the base of the lower baffle.

In order to collect cloud water samples, another collector has also been used, consisting of 0.45 mm diameter Teflon-coated wires, strung at 3 mm intervals around the perimeters of two 25 cm diameter plastic disks, held 1 m apart by plastic rods [21]. The surface area of the collector is sufficient to provide a sample of 50 mL in 3–30 minutes, depending on the wind speed and cloud liquid water content. The collection efficiency of this sampler indicates that cloud droplets of 10 $\mu$m diameter are collected with only 50% efficiency at wind speed of 1 m/s, proving poor performance of the collector at low wind speeds. For 80% of the cloud water samples, the wind speed was greater than 5 m/s, yielding 50% collection diameters below 5 $\mu$m.

### 5. ACTIVE COLLECTORS

Active collectors use either forced flow (fans, pumps) or motors moving the collecting elements in the air (usually rotating them) to achieve the same end. Active collectors typically use collection elements such as rods, tubes, strings, ropes, filaments, screens, and flat surface. Some active collectors use jet-driven impaction onto solid surfaces. Size-resolved cloud composition is usually obtained via active collectors with multiple jet/impaction surface combinations, or multiple stages with varying cutoff diameters.

The active string screen fog sampler described by Jacob et al. [22] consists of three parts: a series of three screens of Teflon wires on which the fog condenses, a baffle which smoothes the airflow, and a fan which pulls the air past the Teflon wires. The fog water is collected on the wires until drops are formed, then they move down the wire, pool in a Teflon tray, and are then collected in a clean glass jar [10]. Inclining the screen at 35 degrees from the vertical in the direction of airflow helps prevent resuspension of impacted droplets into the airflow. During sampling the fog water only comes into contact with Teflon and Glass. During sample collection, the face velocity of the air through the sampler, the time of collection, and the air temperature are monitored. Typical sample collection volumes range from 50 to 200 mL, which can take from 50–180 minutes depending on the liquid water content LWC of the fog event (0.1–0.3 g/m$^3$) [23]. Droplets in the range 3–100 $\mu$m diameter are efficiently collected using this type of sampler [22].

Figure 4 presents a schematic diagram of a typical active string screen collector. The Teflon screen consists of a frame of four 4 mm thick copper rods, over which Teflon strings are strung [24]. Drops collected on the strings find their way to polyethylene collecting bottles. Additional string screen collectors are described by Sasakawa and Uematsu [25] and Sasakawa et al. [26].

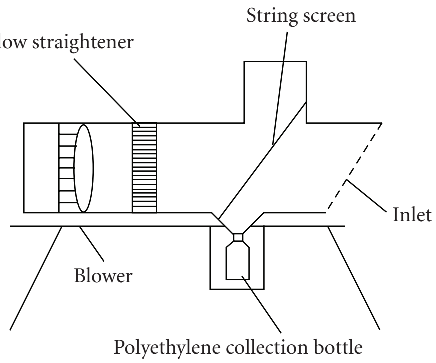

Figure 4: Schematic diagram showing the construction of an active screen fog water collector [24].

The High-Volume Fog Sampler [27, 28] is a scaled-up version of the sampler described by Jacob et al. [22]. In this system, a 50 cm diameter fan in the back draws air at the rate of 4400 m$^3$/h across a screen consisting of four layers of 0.28 mm Teflon filaments wound around threaded rods. Fog droplets impact on the Teflon filaments, coalesce, and flow down the filaments into a Teflon-coated funnel. The fog water then drains by gravity through a Teflon tube to a Teflon bottle. Collection rate of this system is approximately 1 L/h in fog with 400 m visibility.

The Active String Cloud Water Collector (CWP) described by Daube et al. [20] is shown schematically in Figure 5. This system collects cloud droplets on a removable cartridge of 0.78 mm diameter Teflon strands. A fan inside the collector draws air and cloud droplets up through a ventral opening and then into the vertical collection strands. The positioning of the air inlet on the bottom of the sampler makes it possible to avoid collecting rain [29–31]. The collector excludes most rain droplets $\geq 200\,\mu$m at wind speeds $\leq 10$ m/s. With this system, cloud water collection is usually initiated within 15–30 minutes after the onset of a cloud event, and the typical collection time is approximately 5 hours.

The CalTech Active Strand Cloud Water Collector (CASCC), built at California Institute of Technology, is shown schematically in Figure 6. It has been described in detail in several publications [4, 11, 32–35]. In this system, cloud droplets are collected by inertial impaction on an angled bank of six rows of 508 $\mu$m diameter Teflon strands. A fan sucks in air through the Teflon strings with a velocity of 8.5 m/s. The strands are inclined at an angle of 35 degrees from vertical. The collected droplets coalesce, and are drawn down the strands by gravity and aerodynamic drag into a Teflon trough. A Teflon tube delivers the sample from the trough to a collection bottle, which is emptied at 30–60 minutes intervals. The 50% collection efficiency cutoff, based on droplet diameter and predicted from impaction theory, is 3.5 $\mu$m. A protective rain shield, with its opening facing downward, can be attached to the front of the collector to

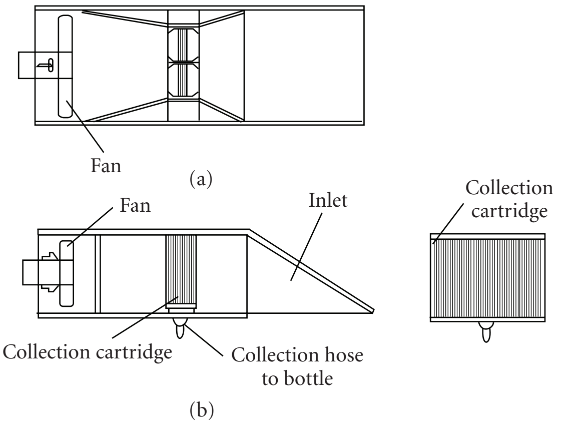

Figure 5: Schematic diagram of an active cloud water collector (CWP): [20] (a) top view, (b) side view.

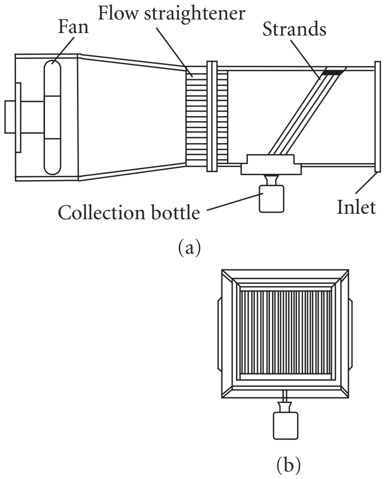

Figure 6: Schematic diagram showing the construction of CASCC collector [32]: (a) side view, (b) front view.

exclude large sedimenting droplets (d > 300 $\mu$m) [34]. The typical flow rate of air through the CASCC is 24.5 $m^3$/min, yielding a collection rate of approximately 2 mL/min when the LWC of the fog is 0.1 g/$m^3$.

The CASCC cannot function below 0°C, because cloud water droplets will freeze on the collection surface. For this reason, a winter cloud water sampler—the CalTech Heated Rod Cloud Water Collector (CHRCC)—was developed [36]. The stainless steel rods that form the collection surface in this sampler are internally heated on a periodic basis when temperatures fall below 4.5°C (Figure 7). When heated, accumulated frozen cloud water on the rods melts and drains off the rods to the sample bottle. A fan draws air across an inclined bank of six rows of the 3175 $\mu$m diameter rods at a rate of 6.3 $m^3$/min. The corresponding cutoff of the rod bank is calculated to be 7.7 $\mu$m. The predicted collection rate in a cloud with an LWC of 0.1 g/$m^3$ is 0.44 g/min.

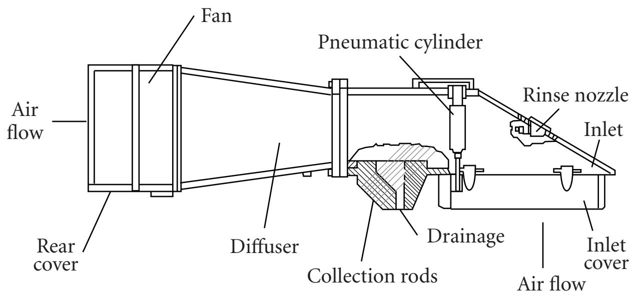

Figure 7: Schematic diagram showing the construction of CHRCC [36].

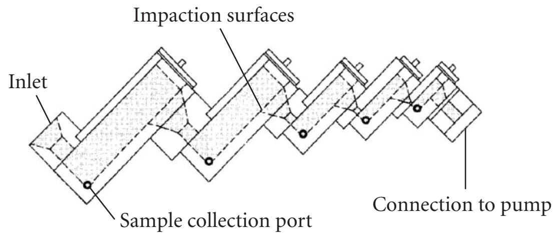

Figure 8: Schematic diagram showing the construction of CSU 5-Stage Collector [37].

### 6. MULTISTAGE FRACTIONING SAMPLERS

The operation of Two-Stage Fog Water Impactor (TFI) [16] is also based on the principle of inertial impaction on a plane surface. This sampler consists of vertical slit impaction stages, one to collect the larger droplets, followed by two identical stages in parallel. These collect those droplets which passed the first stage, but which are above a well-defined cutoff diameter. The cutoff diameters of the first stage are between 10 and 12 $\mu$m, and of the second stages are between 5 and 6 $\mu$m, calculated for flow rates from 150 to 200 $m^3$/h. The air, together with smaller water droplets, leaves the impactor through holes in the plate and four suction pipes. The collected samples are forced by airflow to the external edges and to streams directed down the plates. The samples are collected into vials situated at the bottom of the device. This apparatus has the capability of controlling the velocity of incoming air depending on the average wind speed.

Cloud chemistry can vary as a function of drop size. In order to investigate variations in chemical composition across the drop size spectrum, a multistage cloud water collector was developed by Moore et al. [37] at Colorado State. The CSU 5-Stage Collector (Figure 8) is a cascade inertial impactor that collects samples of cloud water in five independent size fractions for chemical analysis. Its design incorporates many features to facilitate its use in the field, and to maintain both consistent performance between varying atmospheric conditions and the chemical and physical integrity of the collected sample. The sampler consists of five stages, each with a single, one-sided rectangular jet arranged

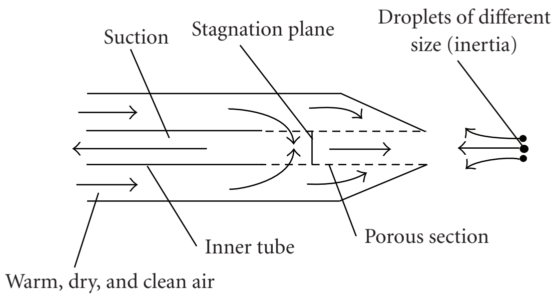

Figure 9: A schematic diagram of CVI sampler.

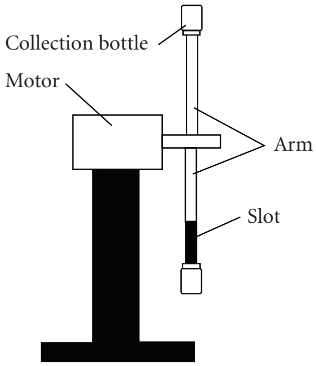

Figure 10: Structure of a fog and cloud water rotating arm collector [11].

in a cascade. The intensity of airflow rises as it passes through the sampler. Drops of progressively smaller diameters are collected in each stage as those with too much inertias cannot follow the fluid streamlines and impact. The collector is mounted at 45 degrees to the horizontal, so sampled drops coalesce and run down to polypropylene vials threaded directly into each stage. The collector is oriented into the wind during operation, subject only to site restrictions and its own geometry. While the collector is designed for low wind environments, a baffle or windshield parallel to the inlet can be added for higher winds. The experimentally determined 50% cutoff diameter for the first stage was 25.5 $\mu$m, while the second stage had a slightly higher 50% cutoff diameter of 29 $\mu$m. Stages three, four, and five had 50% cutoff diameters of 17.5, 10.5, and 4.5 $\mu$m, respectively. Although some mixing between drop sizes occurs, the CSU 5-stage effectively separates the largest drops ($> 30\,\mu$m in diameter) from the smallest ones ($< 10\,\mu$m in diameter) [38].

An important drawback of most cloud and fog water samplers is their inability to separate completely the water particles from the surrounding air. The Counterflow Virtual Impactor (CVI) collector described by Krupa [15] is shown schematically in Figure 9 and offers a solution to this problem. Warm, dry, and particle-free airflows through the annular region of two concentric tubes to the tip of the impactor. The wall of the inner tube at the tip is made of porous stainless steel, which allows the dry air to flow into the inner tube. A fraction of the air entering the inner tube is sucked back into the sampler, while the rest blows out the tip. A stagnation plane, where no net axial flow occurs, is formed inside the porous section of the inner tube. Tipward of this plane, the airflows towards the tip, while inward of this plane, the airflows back into the sampler. The distance from the stagnation plane to the tip can be varied by adjusting the airflow rates to the tip and back into the sampler. This plane is the virtual impaction surface. The device must be moving in the air, tipward (it is frequently airplane-mounted). Cloud droplets approaching the CVI tip can either be deflected around the inlet (smaller ones, low inertia, also small solid particles) or swept into the inner tube (inertia sufficient to reach the stagnation plane). One can adjust the lower size limit of droplets being sampled (from 4 $\mu$m to 15 $\mu$m) by suitable positioning of the stagnation plane. The sampled cloud droplets evaporate quickly in the warm, dry air inside the CVI sampler. The maximum droplet radius that can be sampled is between 50 and 100 $\mu$m. Ambient gases and submicron aerosol particles are rejected in the CVI with almost 100% effectiveness. The instrument requires a condenser for water collection and measurement. In this respect, a CVI collecting sample for chemical analysis must ensure total recovery from the gaseous phase of both water and other volatile substances (pollutants) contaminating original droplets caught by the device.

### 7. ROTATING COLLECTORS

The active sampler described by Glotfelty et al. [6] is a rotating screen device, 50 cm in diameter, in which four layers of stainless steel screen are rotated around a central axis at 720 rpm. Fog water obtained from droplets impacting on the screen is centrifuged to the periphery, collected in a slotted aluminum tube, and drained into a collection vessel. A large fan pulls air through the device at 160 $m^3$/min. Under these conditions, the collection rate of this system is approximately 0.5–1 L/h depending on the LWC of fog water (0.024–0.08 $g/m^3$).

The CalTech, Atmospheric Science Research Center (ASRC),and AeroVironment (AV) instruments are rotating collectors [14], employing external surfaces for the impaction of the droplets.

Jacob et al. [39] built the CalTech system. It is an external impactor that sweeps through the air at a high velocity (1700 rpm) in order to collect large particles. The arm (63 cm long) spins in a vertical plane, driven by a motor (Figure 10). Each end of the arm has a slot milled into its leading edge. Bottles (30 mL) are mounted at the ends of the arm to collect the water that impacts on the slots. Threaded Teflon tubes are screwed on the ends of the arm and extend inside the collection bottles, preventing the collected fogwater samples from running out after the instrument has stopped. Deflectors prevent water that impacts on the solid part of the arm from entering the slot. Small fins are welded to the back of the arm for extra strength. The entire arm is Teflon-coated to prevent chemical contamination and to facilitate cleaning.

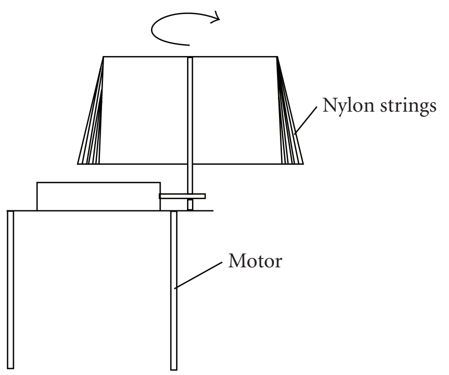

FIGURE 11: Schematic diagram showing the construction of ASRC [14].

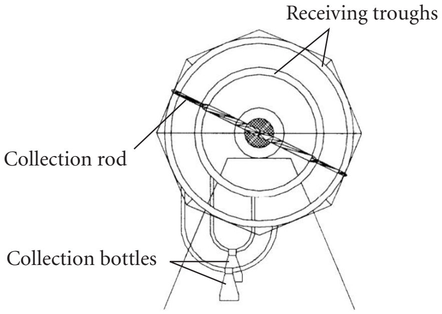

FIGURE 12: Schematic diagram showing the construction of a rotating fog water sampler (AV) [14].

The rotating arm collector samples air at a rate of 5 $m^3$/min. Laboratory calibration indicates a lower size cutoff of 20 $\mu$m diameter (50% collection efficiency). This sampler has been applied in several studies [40, 41].

The ASRC sampler consists of 1 500.41 mm diameter Nylon strings mounted between two plates (Figure 11). The sampler rotates to its vertical axis at 100 rpm [14]. Water impacting on the strings collects in traps on the bottom plate. Periodically, the sample rotation is stopped and the fogwater on the strings is coaxed into the traps by tapping the bottom plate with a mallet. At the end of the sampling period, water in the trap is manually transferred to polyethylene bottles. Two versions of the device exist; one of them is passive and the other is active.

The AV rotating rod sampler collects droplets by impaction on a Teflon-coated rod rotated in a vertical plane at 3450 rpm (Figure 12). The outer part of the rod is 1.6 mm and the inner part is 19 mm in diameter to provide size cuts of 2.5 and 10 $\mu$m, respectively, [14]. Water impacting on the rods is transferred by centrifugal force to circular polyethylene troughs that drain to polyethylene collection bottles. Separate troughs and sample bottles are used for the two size fractions.

In Table 2 parameters of samplers used for collecting fog and cloud were set together.

### 8. OTHER TYPES OF SAMPLERS

The Global Geochemistry Mesh Impaction Fog Sampler and the Desert Research Institute (DRI) instruments are internal collectors [14], in which air is drawn into the instrument and extracted by surfaces internal to the device.

The Global Geochemistry Mesh Impaction Fog Sampler (Figure 13) is an internal impaction sampler that collects fog water on a 10 cm diameter by 4 cm thick polypropylene mesh located at the entrance of a V-shaped Teflon lined PVC pipe. The mesh is made of interlaced filaments (410 $\mu$m diameter) and has a void volume of 96%. Air is drawn through the mesh at 1.7 $m^3$/min. Fog droplets impact on the mesh, coalesce, and then drain into a polyethylene bottle at the bottom of the V-tube. The sampler can effectively intercept droplets $> 5.0\,\mu$m, with a 50% collection efficiency at 2.4 $\mu$m. Liquid holdup on the mesh depends on the mass of liquid sampled. If $\leq$ 1 g of water is sampled, all of it remains on the mesh. If 100 g is sampled, less than 5% remains [14].

The DRI (Figure 14) is based on a jet impaction principle. Fog is drawn through three rectangular jets at a total flow rate of 20 L/s. The accelerated droplets impact on rotating Teflon rollers and are transferred to a central roller. Here, the fogwater is forced to accumulate in bulk form and is deposited into a polystyrene collection vessel. The impactor has a sharp cutoff at 5 $\mu$m diameter to allow efficient collection of droplets while rejecting small interstitial particles. The collector is housed in a shelter consisting of an inverted, insulated 250 l drum to prevent collection of precipitation. Airflow up to the collector is provided by a fan [14, 44].

Specially [8] designed equipment, consisting of a stainless steel cooling chamber (10 cm $\times$ 10 cm $\times$ 22 cm) and a collector for fog droplets utilizing the impaction technique, has also been used for collecting fog water. Fog air is drawn at the rate of 0.2 $m^3$/min and the fog droplets impact on the collector which is maintained at 15°C in the cooling chamber. The sampling period varies from 30 to 120 minutes, and in this time from 5 to 30 mL of fog water is collected. The volume of the sample collected depends on the duration of the fog event.

In the available literature [15, 46] one can find information on an electrostatic precipitation method capable of sampling single cloud or fog drops (Figure 15). The precipitator is based on the corona discharge principle. A copper electrode is placed as a discharge electrode at a distance of 10 cm above an aluminum precipitation electrode. A 25–30 kV voltage is applied to the discharge electrode for 1 second to produce a spray of electrons or negative charge and the charge is transferred to the droplets by the action of the electric field. Charged droplets moving in the direction of the collecting electrode are captured on Petri dishes. The droplets are covered immediately with paraffin oil to prevent evaporation and contamination. Capillary electrophoresis is then used for the chemical analysis of the individual drops. This sampler can also be used for collecting bulk phase cloud or fog water, by increasing the time for the application of the voltage to the discharge electrode from 1 second to 5–15 minutes, depending on the density of the fog.

Table 2: Basic parameters characterizing fog and cloud collectors.

| Name of sampler | Type of sampler | Collector surface | Collection rate (mL/min) | Cutoff ($\mu$m) | LWC (g/m$^3$) | Flow rate (m$^3$/min) | Comments | Reference |
|---|---|---|---|---|---|---|---|---|
| String screen sampler | Active | Teflon wires | 50–200/ 50–180 | 3–100 | 0.1–0.3 | | Inclining the screen at 35 degrees from the vertical in the direction of airflow helps prevent re-suspension of impacted droplets into the airflow. | Jacob et al. [22] |
| High-Volume Fog Sampler | Active | Teflon filaments | 16.7 | | | 4400 | 1 L/h in fog with 400 m visibility, 50 cm diameter fan. | Schomburg et al. [27], Chernyak et al. [28] |
| CWP | Active | Teflon strands, 0.78 mm diameter | | | | | The collector excludes most rain droplets ≥ 200 $\mu$m at wind speeds ≤ 10 m/s. | Daube et al. [20] |
| CASCC | Active | Teflon strands, 508 $\mu$m diameter | 2 | 3.5 | 0.1 | 24.5 | The strands are inclined at an angle of 35 degrees from vertical. | Igawa et al. [4, 33], Munger et al. [32, 34] |
| CHRCC | Active | Stainless steel rods | 0.44 | 7.7 | 0.1 | 6.3 | The stainless steel rods that form the collection surface in this sampler are internally heated on a periodic basis when temperatures fall below 4.5°C. | Collett et al. [36], Schell et al. [16] |
| TFI | Active | | | 5–12 | | 150–200 | This apparatus has the capability of controlling the velocity of incoming air depending on the average wind speed. | Schell et al. [16] |
| CSU 5-Stage Collector | Active | | | 4.5–29 | | | The collector is mounted at 45 degrees to the horizontal. It is a cascade inertial impactor that collects samples in five independent size fractions. | Moore et al. [38] |
| CalTech Rotating Arm Collector | Rotating active | Teflon-coated | | 20 | | 5 | The arm spins in a vertical plane, driven by a motor at 1700 rpm. | Jacob et al. [39, 41], Johnson et al. [40] |
| Rotating screen sampler | Rotating active | Stainless steel screen | 8.3–16.7 | | 0.024–0.08 | 160 | Fifty-centimeter diameter screen rotated around a central axis at 720 rpm. | Glotfelty et al. [6] |
| AV | Rotating active | Teflon-coated rod | | | | | Rod rotated in a vertical plane at 3450 rpm. | Hering et al. [14] |
| ASRC | Rotating | Nylon strings, 0.41 mm diameter | | | | | The sampler rotates to its vertical axis at 100 rpm. Two versions of the device exist, one of them is passive, and the other is active. | Hering et al. [14] |

Table 2: Continued.

| | | | | | | | |
|---|---|---|---|---|---|---|---|
| Global Geochemistry Mesh Impactor | Mesh impaction | Polypropylene mesh | | 2.4 | 1.5–1.7 | | Hering et al. [14], Krupa [15] |
| Sampler with cooling chamber | | Stainless steel cooling chamber | 5–30/ 30–120 | | 0.2 | The volume of the sample collected depends on the duration of the fog event. | Khemani et al. [8] |
| Passive collector | Passive | Nylon strings, 0.2 mm diameter | | | | Collection area 314 $m^2$. | Lange et al. [19] |
| AMC/WPI | Passive | Teflon strands | | | | Baffle system. | Daube et al. [20] |
| Passive sampling system | Passive | Teflon strings, 0.3 mm diameter | 15–25/120 | | | The sampler is activated and closed by a fog sensor based on the dew point and a separate rain sensor. | Krupa [15] |
| Automated sampling system | | Teflon strings | | | | The measurement of pH and conductance occurs automatically in real time during sampling. | Baumgardner et al. [3], Mohnen et al. [42], Vong et al. [43] |
| CRAC | | | | 2 | | The instrumentation and the electronic section include a conductometer, a pH meter and a microprocessor. | Jacob et al. [22] |

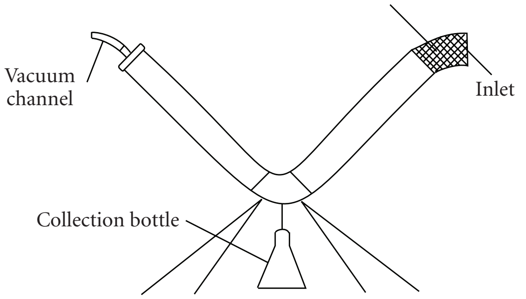

Figure 13: Construction scheme of a fog water sampler (Global Geochemistry Mesh Impaction) [15].

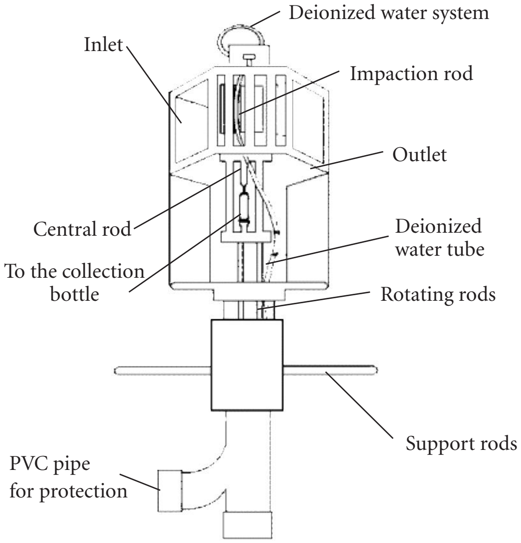

Figure 14: A schematic diagram of DRI sampler [45].

### 9. AUTOMATIC DEVICE

Figure 16 provides an example of a passive sampling system [15]. It consists of a Teflon support structure and 0.3 mm diameter Teflon strings, mounted 3.0 mm apart in a cylindrical configuration. Under appropriate airflow conditions, fog droplets are impacted on these strings, grow to larger drops, run down the strings, and are collected into bottles. All droplets $> 5.0\,\mu$m diameter are impacted at normal wind speeds. In order to collect 15–25 mL it is necessary to collect samples for two hours. This collector is set out only in case of the occurrence of fog. In other situations it is enclosed inside a metal cylinder in order to prevent its contamination (rain and dry deposition). The sampler is activated and closed by a fog sensor based on the dew point and a separate rain sensor. A modified dynamic version of the passive sampler is the CalTech Active Strand Cloud Water Collector.

An automated system for the collection of cloud water samples directly from clouds (Figure 17) has been described in various papers [3, 42, 43]. The system consists of a collector which uses wind speed to affect cloud impaction on 0.4 mm Teflon strings, a system for collecting, retaining, and storing samples, and an electronic unit controlling the system. The equipment includes also a temperature sensor, a rain detector, and a device for measuring wind speed. When there are no clouds, it is stored within a protective enclosure. During cloud events, a motor-driven shaft elevates and exposes the collector. The sample storage unit

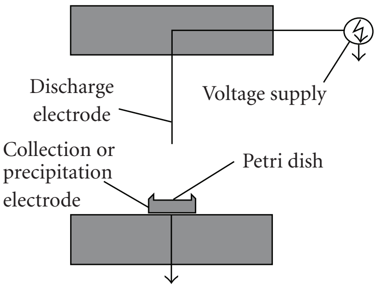

Figure 15: Structure of a device for electrostatic precipitator for sampling single cloud or fog water droplets [15].

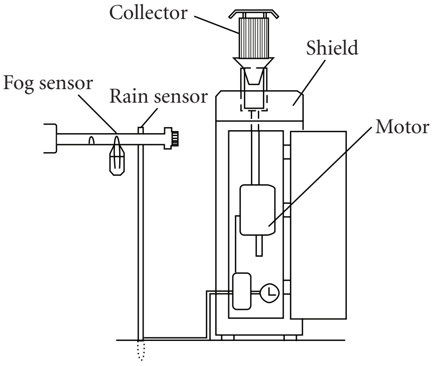

Figure 16: Structure of a passive fog water sampling system [15].

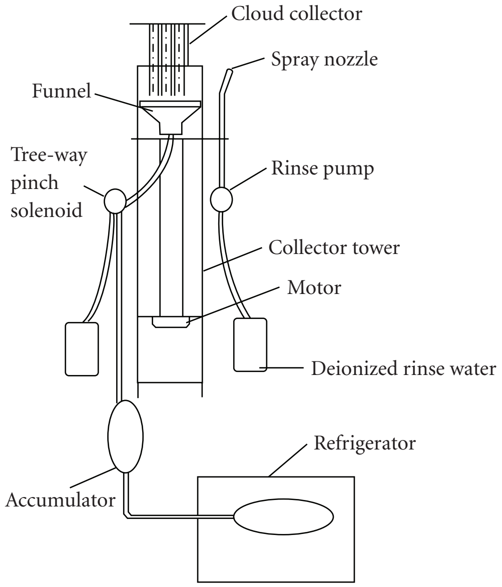

Figure 17: Structure of an automatic system to collect rain water samples directly from the clouds [3].

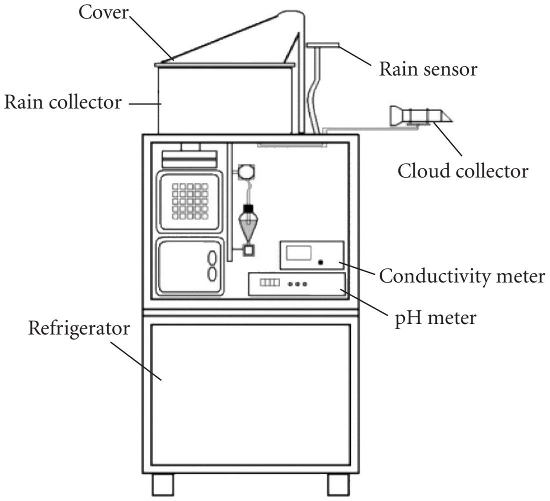

Figure 18: Structure of an automatic system for the analysis of cloud water samples [5].

consists of 24 l polyethylene sample bottles contained in a circular wire support and housed in a commercial refrigerator. When the liquid water content of a cloud exceeds 0.05 g/m$^3$, the wind speed is higher than 2.5 m/s, ambient air temperature is above freezing, and there is no rainfall, the cloud water collector is activated and projected out of its protective housing. The measurement of pH and conductance occurs automatically in real time during sampling. The indications of pH values are checked on a daily basis and the conductance value is indicated in relation to the external temperature.

An automated system for collecting and analyzing rain samples (Cloud and Rain Acidity/Conductivity Analyzer (CRAC)) has been adapted for the analysis of cloud water [5]. The system consists of a rain probe connected to an active CalTech collector [22] (Figure 18). When cloud water samples are being collected, the rain detector is disconnected. The instrumentation and the electronic section include a conductometer, a pH meter, and a microprocessor. Samples are collected sequentially in double accumulation vessels containing conductometric cells. After having collected 50 mL of samples, the system directs 12 mL to a chamber where pH is measured, while the remaining quantity goes to a vial which is kept in an automatic whirling arm. The whirling arm and the pH meter are situated in the cooling section. This collector has a 50% cutoff size centered around 2 $\mu$m diameter.

In published papers [47, 48], an automatic device for collecting fog samples has been described. Figure 19 shows a block diagram of this instrument [48, 49]. The microprocessor, after receiving signals from three sensors: fog, temperature, and rain, uses them to control the system of fog droplet collection. The fog detector, being an optical backscattered sensor (Figure 20), consists of two receivers; one of which measures the intensity of the light source and the other the intensity of the light scattered by the fog droplets. Both signals reach the microprocessor and their ratio is compared with a threshold value. The system is activated when the detector signals the presence of fog. An active string collector was used to collect fog samples because of its simple structure and ease of automation. This collector consists of a polyethylene aerodynamic tunnel in which air is sucked in by

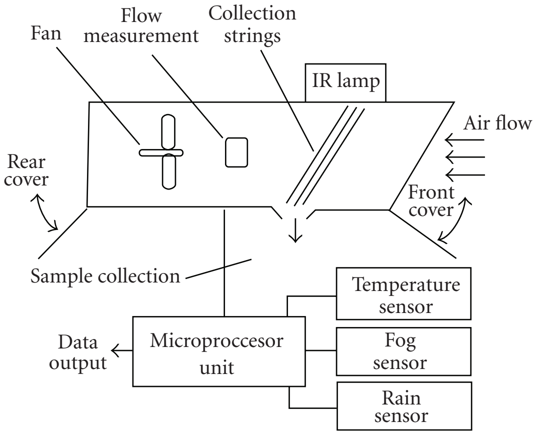

FIGURE 19: Block diagram of the automatic instrumentation for fog water sampling [48].

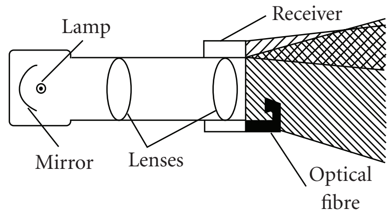

FIGURE 20: Schematic diagram of the fog optical detector [47].

a fan located in its rear part. Teflon strings (0.4 mm diameter) are strung 5 mm apart from each other in the form of a vertical shield, on three frames placed at an angle of 30 degrees with respect to the direction of the stream. Fog droplets collide with them and once they reach an adequate size, they flow down into bottles. The velocity of airflowing through the tunnel is 6 m/s, which corresponds to an airstream intensity of 17 $m^3$/min. The collector opens and closes automatically. At the end of fog occurrence the lid is closed in order to keep away impurities. In order to prevent the freezing of fog droplets on the strings, an infrared emitting lamp is placed above the frames, which allows the hoarfrost on the strings to melt into the collecting bottles. A simplified version was used in [50].

### 10. COLLECTING DEW SAMPLES

Dew typically forms on cool nights with light breezes; water condenses from the atmosphere at ground level under these conditions. Hence, ground effects control the atmospheric chemistry during condensation [51]. Dew can play an important role in the deposition of air pollution particularly in arid ecosystems [18]. Dew may increase seedling survival, plant growth, and crop yield, but it may also have a negative effect, promoting bacteria and fungal infections. As a source of information on the environment, dew samples have long been a subject of study [52]. During the night, the latent heat flux towards the soil surface is very small, and therefore the amounts of dew deposition are very small as well. This fact poses special technical measurement difficulties that we will discuss in the following paragraphs.

The first publications presenting methods for determining the amount of dew appeared at the end of the last century [52]. Measurements were carried out using very simple methods, that is, collecting dew from grass by means of a sponge or by placing absorbent paper on grass. The dew samples were collected early in the morning. Before the expected appearance of dew, the collecting surface was flushed with deionized water and subsequently dried. Dew collection took place only on rainless nights to eliminate any influence of rain droplets on collected dew samples.

Modern methods of dew collection can be divided into three basic groups: optical, volumetric, and gravimetric. Modern methods permit continuous registration of dew events in their nascent stage, measurement of amount of dew, its duration, and evaporation time. Optical methods consist in visually estimating the amount of dew. In this method the device Duvdevan is often used.

Volumetric methods are connected with measuring the amount (volume) of the collected dew. Volumetric methods are exemplified by the volumetric drosometer, which has a collecting surface consisting of a filter paper 9 cm in diameter, saturated with water [53]. This instrument has limitiations: it can be used only at temperatures above 0°C and the exact moment of dew formation must be determined.

Gravimetric methods rely on defining the increase of the weight of the collecting surface (without and with dew), which can be determined by means of analytical balance. Gravimetric methods include the method using Leick's plates (made from a mixture of silicon dioxide dust, alabaster gypsum, and water) [53]. The plates are weighed before being used and after they are bedewed. The increase in weight in mg is proportional to the amount of dew in mm. Gravimetric methods include the drosograph too. The nascent dew settles upon a receiving plate and then flows down to a collecting vessel located underneath. The plate and vessel are located at one end of a first-order lever, the opposite end of which ends in a small writing pen. The movement of the pen, combined with the rotating movement of the drum, makes it possible to record changes in the quantity of dew. The drosograph described by Hutorowicz [54] uses an analytical balance with a metal box with turf in the role of dew collector and holder.

Li [55] has described another direct gravimetric technique. The collectors are containers made of polymethylmethacrylate (PMMA), with a diameter of 9.2 cm and a height of 10 cm, filled with an appropriate collecting material: thick gravel, sand, and/or loess.

As with cloud and fog, dew can deposit atmospheric pollution and in recent years, interest has grown in dew sampling for analysis of atmospheric pollution. The dew sampling method most often used for experiments on atmospheric pollution is the cloth plate method (CPM) [18, 56], a variant of the gravimetric method of dew collection. Velvet-like fabric (a square cloth of 6 cm × 6 cm), 0.15 cm thick, is placed in the centre of a 10 cm × 10 cm × 0.2 cm glass plate.

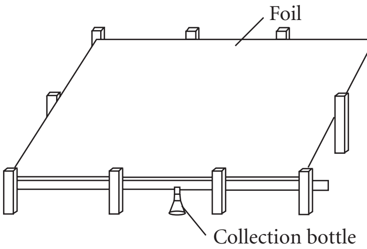

Figure 21: Schematic diagram of a dew sampler [57].

The glass plate is placed on a 10 cm × 10 cm × 0.5 cm layer of plywood. The plate and the plywood form a homogenous base 0.7 cm thick. The absorbent material is removed and replaced every day. Upon removal it is placed in flasks and weighed in order to measure dampness.

In the collector described in paper [57] and shown in Figure 21, the condensation surface is a rectangular foil sheet, 3 m × 10 m, made from $TiO_2$ and $BaSO_4$ microspheres embedded in polyethylene (volumetric method). The foil is fixed by lateral cables on a light grid attached by cables. The cables are fixed to beams anchored to the ground. This foil exhibits improved emitting properties in the near infrared (to provide radiative cooling of room temperature surfaces) and efficiently reflects the visible radiation (sun). A weak wind (< 1 m/s) is necessary to provide sufficient humid air around the condenser, but strong wind increases heat losses. To minimize wind influence and recover water drops by gravity using a plane condensing area with an angle with respect to horizontal and thermally isolated from the ground with 2 cm thick polystyrene foam, the placement of the collector at an angle of 30 degrees facilitates the flow of droplets (it seems that the angle may be a critical value, too high leads to diminishing dew formation). Dew, accumulating in a groove along the lower edge of the collecting plane, flows off to a 25 l polyethylene bottle.

Dew has been sampled by means of a collector which consists of a pump and a Teflon pipe terminated with a glass fibre filter [64]. The dew is sucked in by the pump together with air and flows through the pipe into a polyethylene bottle. Within 30 minutes, 1 mL of dew is collected.

In Table 3 parameters of samplers used for collecting dew were set together.

### 11. COLLECTING HOARFROST/RIME SAMPLES

Rime and hoarfrost are physical processes and forms when supercooled cloud droplets in a basal cloud layer freeze on impact with vegetation or topographic surfaces. Rime is commonly observed on trees, towers, power lines, and other objects at high elevations exposed to high-velocity cloud airflow. The difference between rime and hoarfrost was explained in Table 1. The chemical characteristics of rime, hoarfrost, and glaze should mimic the chemistry of cloud droplets and potentially affect vegetation adversely. If these deposits act as a biologically inert, frozen protective shell, their role could be beneficial. Hoarfrost and rime constitute an important element in water circulation, particularly in mountainous regions where they occur quite frequently, they contribute to the process of cleaning the atmosphere and of transferring impurities from air to the soil. Such collectors can be used interchangeably because the precipitation depends on meteorological conditions.

The simplest samplers used to collect samples of hoarfrost and rime consist of flat surfaces made from materials such as Teflon, galvanized steel [79], or Nylon wires [80]. Very often hoarfrost accumulates on some elements of cloud water collectors, for example, the strings, and it is then collected manually into polyethylene bottles [30, 81]. In Table 4 parameters of samplers used for collecting rime were set together.

In another published paper [60] an apparatus was described as being consisting of four polyethylene plates. The plates are fastened at the height of 1 m on an aluminium scaffold and positioned vertically facing the four cardinal points. The wind direction is estimated during sample collection by observing the wind vanes. Hoarfrost or else rime which accumulates on the surface of the plates is removed by means of a polyethylene scraper from the outer sides of the plates and collected into bottles.

For collecting samples of hoarfrost and rime, a passive collector is used with a string polyethylene screen. The collector mesh has 12 mm openings with 2 mm strands. Samples gathering on the screen are collected into 250 mL containers [61].

In the passive shield hoarfrost/rime collector is made of polyvinyl chloride and the collecting area consists of 46-singular fibres 0.2 mm in diameter. Deposit is scraped off from its surface by means of a scraper. Subsequently, the samples are placed in polyethylene bottles and transported to the laboratory [62].

To measure liquid and solid atmospheric deposits, the instrument known as the Grunow thimble is used also. It consists of a wire mesh in the form of a cylinder with a diameter of 10 cm and a height of 20 cm [82, 83]. This thimble is laid over a rain gauge, for example, of the Hellman type (the instrument consists of a metal cylinder ending in a funnel and the precipitation—depending on its kind—flows down into the container or accumulates above the funnel).

### 12. AUTOMATIC DEVICE FOR SOLID DEPOSITS

Measurements of solidified atmospheric deposits (hoarfrost, rime, freezing rain, and glazed frost) can be of substantial significance for certain branches of economy, such as the power industry, or air and road transport. It must be said that some problems may arise to differentiate between solid deposited precipitation and freezing fallen precipitation (freezing rain), both contributing to the icing events. A sufficiently thick layer of the deposit can overload and possibly break electric power lines. In a published paper [84] a method is presented for measuring the process of accretion of solid deposits on specific surfaces. The method relies on the measurement of the weight of ice deposits, the visual description of their appearance, and the time and duration of their occurrence. For this purpose, several pairs of electric power

Table 3: Basic parameters characterizing dew collectors.

| Type of collector surface | Dimension (mm × mm) | Thickness of collector base (mm) | Comments | Reference |
|---|---|---|---|---|
| PTFE-sheet | 310 × 307 | 3 | The collectors were placed on a 10 cm block of polystyrene foam. | Takenaka et al. [58] |
| Pyrex glass plate | 300 × 298 | 3 | | |
| Stainless steel sheet | 303 × 228 | 0.5 | | |
| Aluminium sheet | 303 × 226 | 1 | | |
| PTFE-coated stainless steel sheet | 303 × 228 | 0.5 | | |
| PTFE-coated aluminium sheet | 303 × 226 | 1 | | |
| Plastic foil | 2000 × 2000<br>2500 × 2500 | 0.05 | Dew drops form on surfaces slightly inclined down the foil, they are collected in the morning by means of a syringe and put into 100 and 250 mL bottles. | Scheller [59] |
| Glass | 1000 × 1000 | 1 | In order to insure that dew will form, the back of the collector is made of aluminium. | Jiries [51] |

Table 4: Basic parameters characterizing RIME collectors.

| Type of collector surface | Dimension ($cm^2$) | Location | Comments | Reference |
|---|---|---|---|---|
| Polyethylene plates | 400 | At the height of 1 m | It is also possible to measure the size of the collected deposit and to record its visual appearance by photographing it. | Ferrier et al. [60] |
| Polyethylene screen | 3600 | At the height of 2–3 m | Deposits are collected for the whole 24-hour period. | Berg et al. [61] |
| Polyvinyl chloride shield | 929 | At the height of 1 m | | Duncan [62] |
| Teflon film | | | Teflon film attached to a 3 cm thick polystyrene block with double-stick tape. | Foster [63] |

line sections of different diameters are used. A single measurement set consists of four conductor sections of a specific diameter and length, suspended in pairs at some set height above the ground. During the observation, the thickness of the deposit on the conductors is measured with a pair of calipers. When the thickness exceeds 10–20 mm, both conductors are taken down and transferred indoors for the purpose of melting and weighing, or measuring the volume of water by means of a laboratory-graduated cylinder. A published paper [85] presents an installation for the measurement of frosting on power lines.

Measurement methods are usually based on the determination of the weight of hoarfrost (icing) per unit of area or length (the latter value is important for the power industry). This weight may be determined by means of a device [86] which consists of two wooden rods, where frost/ice formation occurs, laid perpendicular to each other; the first pointing north-south and the other east-west. The modified version of the device utilizes a strain-gage beam force sensor connected to the cylinder collecting the ice.

In [84] a system is presented which is used to measure frosting. It consists of continuously operating vibrational sensors of freezing rain and frosting. The sensor is a small cylindrical metal core, electromagnetically excited to vibrate at a nominal resonance frequency of 40 kHz. Two feedback coils covibrating with the core, placed in it, permit the measurement of the actual frequency of core vibrations through a microprocessor-based measuring and control circuit. When frosting (freezing rain, rime, and hoarfrost) starts to form on the core, the mass of the vibrating object increases, which leads to a proportional reduction of the frequency of core vibration. When the frosting reaches a thickness of 3.8 mm, heating the sensor is switched on automatically in order to melt the deposit and to restore the resonant frequency. Periods of defrosting and renewed frosting build-up last for 5–10 minutes, depending upon the wind speed and temperature of

Table 5: Information on the concentrations of inorganic and organic pollutions in fog, cloud, dew, and rime samples.

| Sampler | Atmospheric **precipitation or deposits** | Analytes | Concentration range (mg/L) | **Reference** |
|---|---|---|---|---|
| CalTech Rotating Arm Collector | Fog | $NO_3^-$ | 0.37–489.8 | Jacob et al. [41] |
| | | $SO_4^{2-}$ | 2.59–99.84 | Johnson et al. [40] |
| | | $Cl^-$ | 0.67–194.9 | Lacaux et al. [65] |
| | | $NH_4^+$ | 0.77–51.48 | |
| | | $Ca^{2+}$ | 0.6–39.0 | |
| | | $K^+$ | 0.08–5.77 | |
| | | $Mg^{2+}$ | 0.11–41.25 | |
| | | $Na^+$ | 0.23–139.4 | |
| | | pH | 2.16–6.17 | |
| | | Formaldehyde | 0.50–2.30 | |
| | | Acetaldehyde | 0.007–0.17 | Grosjean and Wright [66] |
| | | Benzaldehyde | 0.08–0.32 | |
| | Cloud | $NO_3^-$ | 9.98–1010.6 | Waldman et al. [12] |
| | | $SO_4^{2-}$ | 6.14–446.4 | Grosjean and Wright [66] |
| | | $Cl^-$ | 0.53–343.1 | |
| | | $NH_4^+$ | 1.11–133.6 | |
| | | Fe | 0.02–6.88 | |
| | | Pb | 0.038–2.78 | |
| | | pH | 2.06–3.87 | |
| | | Formaldehyde | 0.01–1.08 | |
| | | Acetaldehyde | 0–0.59 | |
| | | Benzaldehyde | 0–0.57 | |
| | | 2-butanone | 0–0.47 | |
| | | n-butanal | 0–0.52 | |
| CASCC | Fog | $NO_3^-$ | 4.72–1791.8 | Munger et al. [32] |
| | | $SO_4^{2-}$ | 0.013–1196.16 | Klemm et al. [67, 68] |
| | | $Cl^-$ | 0.0027–86.51 | Collett et al. [69] |
| | | $NH_4^+$ | 0.38–464.4 | Anastasio et al. [70] |
| | | $Na^+$ | 0.21–44.48 | Wrzesinsky et al. [71] |
| | | $K^+$ | 0.23–2.67 | Herckes et al. [35, 72] |
| | | Al | 14–903 $\mu$g/L | |
| | | Ni | 0.8–42.8 | |
| | | Pb | 3.4–61.4 | |
| | | Cd | 0.3–8.7 | |
| | | Cu | 2.5–56.8 | |
| | | Sb | 0.8–5.7 | |
| | | Se | 1.1–11.5 | |
| | | pH | 2.33–7.43 | |
| | | Conductivity | 17–452 $\mu$S/cm | |
| | | n-Alcanes | NW-5.1 ng/mL | |
| | | Naphthalene | NW-0.1 | |
| | | Phenanthrene | 0.028–0.169 | |
| | | Anthracene | NW-0.040 | |
| | | Fluoranthene | 0.002–0.095 | |
| | | Pyrene | 0.013–0.289 | |
| | | Benzo(ghi)fluoranthene | NW-0.019 | |
| | | Benz(a)anthracene | NW-0.022 | |
| | | Benzo(e)pyrene | NW-0.252 | |
| | | Benzo(a)pyrene | NW-0.218 | |
| | | Indeno(1.2.3-cd)pyrene | NW-0.007 | |
| | | Benzo(ghi)perylene | NW-0.033 | |

Table 5: Continued.

| | | | | |
|---|---|---|---|---|
| | Cloud | $NO_3^-$ | 1.74–507.8 | Munger et al. [34] |
| | | $SO_4^{2-}$ | 36.48–1112.8 | Anastasio et al. [70] |
| | | $Cl^-$ | 0.39–74.4 | Collett et al. [73] |
| | | $NH_4^+$ | 0.79–168.9 | Igawa et al. [33] |
| | | $Ca^{2+}$ | 0.24–91.4 | |
| | | $Mg^{2+}$ | 0.11–126.6 | |
| | | $Na^+$ | 0.32–87.3 | |
| | | pH | 2.42–4.98 | |
| | | Formaldehyde | 0.41–1.84 | |
| | | Acetaldehyde | 0.053–0.22 | |
| ASRC | Fog | $NO_3^-$ | 0.22–23.15 | Schemenauer et al. [74] and Schemenauer and Cereceda [75] |
| | | $SO_4^{2-}$ | < 0.5–36.4 | |
| | | $F^-$ | < 0.1–1.48 | |
| | | $Cl^-$ | < 0.1–27.9 | |
| | | $Ca^{2+}$ | < 0.10–11.10 | Gordon et al. [9] |
| | | $K^+$ | < 0.08–0.93 | Eckardt et al. [76] |
| | | $Mg^{2+}$ | 0.02–1.79 | |
| | | $Na^+$ | 0.08–15.7 | |
| | | $NH_4^+$ | 0.32–3.19 | |
| | | Al | 0.02–1.56 | |
| | | B | < 0.07–0.22 | |
| | | Cd | < 0.002 | |
| | | Cr | < 0.01 | |
| | | Cu | < 0.01 | |
| | | Fe | < 0.02–0.86 | |
| | | Mn | < 0.003–0.236 | |
| | | Ni | < 0.01 | |
| | | Pb | < 0.03–0.27 | |
| | | Zn | < 0.01–1.03 | |
| | | $HCO_3^-$ | 0.31–1.86 | |
| | | pH | 3.46–6.694 | |
| | Cloud | $NO_3^-$ | 0.70–12.3 | Schemenauer and Cereceda [77] |
| | | $SO_4^{2-}$ | 0.45–6.70 | |
| | | $Cl^-$ | 5.85–84.3 | |
| | | $NH_4^+$ | 0.00–0.65 | |
| | | $Ca^{2+}$ | 2.08–42.7 | |
| | | $K^+$ | 0.14–2.12 | |
| | | $Mg^{2+}$ | 0.46–5.73 | |
| | | $Na^+$ | 3.36–46.0 | |
| | | Fe | < 0.06 | |
| | | Cd | < 0.0005 | |
| | | Pb | < 0.0005 | |
| | | Be | < 0.0005 | |
| | | Cr | < 0.005 | |
| | | Mn | 0.0054–0.034 | |
| | | Ni | < 0.002–0.0074 | |
| | | Cu | < 0.005 | |
| | | Zn | < 0.002–0.026 | |
| | | pH | 6.96–7.94 | |
| High-Volume Sampler | Fog | Diazinon | 0.15–4.8 $\mu$g/L | Schomburg et al. [27] |
| | | Malathion | 0.14–8.7 | Chernyak et al. [28] |
| | | Chlorpyrifos | 0.012–0.19 | |
| | | Fonofos | < 0.012–0.030 | |
| | | Methidathion | 0.036–0.22 | |
| | | Chlorpyrifos | < 900–5000 | |

Table 5: Continued.

| | | | | |
|---|---|---|---|---|
| | | Chlorothalonil | 4000–17000 | |
| | | Endosulphan | 500–10000 | |
| | | Metolachlor | 1800–147000 | |
| | | Trifluralin | 100–1000 | |
| CWP | Fog | $NO_3^-$ | 0.248–284.0 | Vermeulen et al. [29] |
| | | $SO_4^{2-}$ | 0.528–163.2 | Elias et al. [30] |
| | | $F^-$ | 0.02–0.67 | Kimball et al. [31] |
| | | $Cl^-$ | 0.60–24.7 | |
| | | $Ca^{2+}$ | 0.02–16.0 | |
| | | $Na^+$ | 0.09–8.33 | |
| | | $K^+$ | 0.12–19.5 | |
| | | $Mg^{2+}$ | 0.06–5.5 | |
| | | $NH_4^+$ | 0.054–80.5 | |
| | | $Al^{3+}$ | 0.04–1.16 | |
| | | $Li^{3+}$ | 1.00–2.00 | |
| | | $Sr^{2+}$ | 0.03–0.05 | |
| | | pH | 2.41–6.36 | |
| | | Conductivity | 23.2–854 $\mu$S/cm | |
| | Rime | $NO_3^-$ | 0.30–17.9 | Elias et al. [30] |
| | | $SO_4^{2-}$ | 0.70–48.8 | |
| | | $F^-$ | 0.02–0.98 | |
| | | $Cl^-$ | 1.47–28.6 | |
| | | $Zn^{2+}$ | 0.01–1.49 | |
| | | $Mn^{2+}$ | 0.005–0.184 | |
| | | $Mg^{2+}$ | 0.02–1.56 | |
| | | $Na^+$ | 0.07–17.6 | |
| | | $K^+$ | 0.10–9.14 | |
| | | $Ca^{2+}$ | 0.06–7.38 | |
| | | $NH_4^+$ | 0.02–12.5 | |
| | | $Fe^{2+}$ | 0.02–0.57 | |
| | | $Al^{3+}$ | 0.02–1.29 | |
| | | $Sr^{2+}$ | 0.03–0.05 | |
| | | pH | 3.47–6.87 | |
| | | Conductivity | 6.65–252 $\mu$S/cm | |
| Passive collector (polyvinyl chloride) | Rime | $SO_4^{2+}$ | 0.88 | Duncan [62] |
| | | $Cl^-$ | 0.65 | |
| | | $Na^+$ | 0.29 | |
| | | $K^+$ | 0.14 | |
| | | $Mg^{2+}$ | 0.06 | |
| CPM polyethylene | Dew | $Ca^{2+}$ | 0.21 | Muselli et al. [57] |
| | | $NH_4^+$ | 0.31 | |
| | | $NO_3^-$ | 0.77 | |
| | | pH | 5.69 | |
| | | $SO_4^{2-}$ | 0–375 | |
| | | $Cl^-$ | 0–2275 | |
| | | $Ca^{2+}$ | 0–90 | |
| | | $K^+$ | 0–30 | |
| | | pH | 4.4–7.3 | |
| CPM Teflon | Dew | $NO_2^-$ | 2.16–8.14 | Rubio et al. [78] |
| | | $NO_3^-$ | 5.77–14.76 | |
| | | $SO_4^{2-}$ | 8.5–43.25 | |
| | | $Cl^-$ | 0.99–3.05 | |
| | | $NH_4^+$ | 5.68–14.06 | |

Table 5: Continued.

| | |
|---|---|
| $Ca^{2+}$ | 7.54–13.46 |
| $K^{+}$ | 0.27–3.12 |
| $Mg^{2+}$ | 0.69–2.83 |
| $Na^{+}$ | 0.34–2.94 |
| pH | 5.4–6.6 |

the environment. Once every minute a signal appears at the output of the sensor indicating the frequency of vibrations of the sensor.

### 13. MEASUREMENT RESULTS

In the recent years an increased interest is observed in the chemistry of atmospheric precipitation and deposits, as the impurities and pollutants undergo complicated chemical and biochemical reactions in the aquatic and soil ecosystems due to which they enter into biogeochemical circulation, disturbing the environmental balance. For this reason, the pollution of atmospheric air, as well as the pollution of atmospheric precipitation and deposits which follow, constitutes a problem on an international scale, requiring constant monitoring as being confirmed by national and foreign studies collected through literature research. In Table 5 results of measurements of inorganic and organic compound concentrations determined in nontypical samples are presented.

### ACKNOWLEDGMENTS

The authors acknowledge the generous support by the Department of Analytical Chemistry constitutes the Centre of Excellence in Environmental Analysis and Monitoring, which is a research project supported by the European Commission under the Fifth Framework Programme and contributing to the implementation of the Key Action "Sustainable Management and Quality of Water" within the Energy, Environment and Sustainable Development (Contract no. EVK1-CT-2002-80010).

### REFERENCES

[1] M. Grynkiewicz, Ż. Polkowska, and J. Namieśnik, "Pobieranie próbek opadów atmosferycznych do analizy. Opis stosowanych próbników," *Chemia i Inżynieria Ekologiczna*, vol. 9, no. 8, pp. 853–867, 2002.

[2] J. Namieśnik and Z. Jamrógiewicz, *Fizykochemiczne metody kontroli zanieczyszczeń środowiska*, Wydawnictwo Naukowo-Techniczne, Warszawa, Poland, 1998.

[3] R. E. Baumgardner, K. G. Kronmiller, J. B. Anderson, J. J. Bowser, and E. S. Edgerton, "Development of an automated cloud water collection system for use in atmospheric monitoring networks," *Atmospheric Environment*, vol. 31, no. 13, pp. 2003–2010, 1997.

[4] M. Igawa, K. Matsumura, and H. Okochi, "High frequency and large deposition of acid fog on high elevation forest," *Environmental Science & Technology*, vol. 36, no. 1, pp. 1–6, 2002.

[5] V. P. Aneja, C. S. Claiborn, R. L. Bradow, R. J. Paur, and R. E. Baumgardner, "Dynamic chemical characterization of montane clouds," *Atmospheric Environment. Part A. General Topics*, vol. 24, no. 3, pp. 563–572, 1990.

[6] D. E. Glotfelty, M. S. Majewski, and J. N. Seiber, "Distribution of several organophosphorus insecticides and their oxygen analogs in a foggy atmosphere," *Environmental Science & Technology*, vol. 24, no. 3, pp. 353–357, 1990.

[7] M. Millet, H. Wortham, and Ph. Mirabel, "Solubility of polyvalent cations in fogwater at an urban site in Strasbourg (France)," *Atmospheric Environment*, vol. 29, no. 19, pp. 2625–2631, 1995.

[8] L. T. Khemani, G. A. Momin, P. S. Prakasa Rao, P. D. Safai, and P. Prakash, "Influence of alkaline particulates on the chemistry of fog water at Delhi, north India," *Water, Air, & Soil Pollution*, vol. 34, pp. 183–189, 1987.

[9] C. A. Gordon, R. Herrera, and T. C. Hutchinson, "Studies of fog events at two cloud forests near Caracas, Venezuela—II. Chemistry of fog," *Atmospheric Environment*, vol. 28, no. 2, pp. 323–337, 1994.

[10] P. D. Capel, C. Leuenberger, and W. Giger, "Hydrophobic organic chemicals in urban fog," *Atmospheric Environment. Part A. General Topics*, vol. 25, no. 7, pp. 1335–1346, 1991.

[11] J. L. Collett Jr., B. C. Daube Jr., J. W. Munger, and M. R. Hoffmann, "A comparison of two cloudwater/fogwater collectors: the rotating arm collector and the caltech active strand cloudwater collector," *Atmospheric Environment. Part A. General Topics*, vol. 24, no. 7, pp. 1685–1692, 1990.

[12] J. M. Waldman, J. W. Munger, D. J. Jacob, and M. R. Hoffmann, "Chemical characterization of stratus cloudwater and its role as a vector for pollutant deposition in a Los Angeles pine forest," *Tellus. Series B: Chemical and Physical Meteorology*, vol. 37, pp. 91–108, 1985.

[13] K. Plessow, K. Acker, H. Heinrichs, and D. Möller, "Time study of trace elements and major ions during two cloud events at the Mt. Brocken," *Atmospheric Environment*, vol. 35, no. 2, pp. 367–378, 2001.

[14] S. V. Hering, D. L. Blumenthal, R. L. Brewer, et al., "Field intercomparison of five types of fog water collectors," *Environmental Science & Technology*, vol. 21, no. 7, pp. 654–663, 1987.

[15] S. V. Krupa, "Sampling and physico-chemical analysis of precipitation: a review," *Environmental Pollution*, vol. 120, no. 3, pp. 565–594, 2002.

[16] D. Schell, R. Maser, W. Wobrock, et al., "A two-stage impactor for fog droplet collection: design and performance," *Atmospheric Environment*, vol. 31, no. 16, pp. 2671–2679, 1997.

[17] V. A. Marple and K. Willeke, "Impactor design," *Atmospheric Environment*, vol. 10, pp. 891–896, 1976.

[18] G. J. Kidron, "Altitude dependent dew and fog in the Negev Desert, Israel," *Agricultural and Forest Meteorology*, vol. 96, no. 1, pp. 1–8, 1999.

[19] C. A. Lange, J. Matschullat, F. Zimmermann, G. Sterzik, and O. Wienhaus, "Fog frequency and chemical composition of fog water—a relevant contribution to atmospheric deposition in the eastern Erzgebirge, Germany," *Atmospheric Environment*, vol. 37, no. 26, pp. 3731–3739, 2003.

[20] B. C. Daube Jr., K. D. Kimball, P. A. Lamar, and K. C. Weathers, "Two new ground-level cloud water sampler designs which reduce rain contamination," *Atmospheric Environment*, vol. 21, no. 4, pp. 893–900, 1987.

[21] J. A. Ogren and H. Rodhe, "Measurements of the chemical composition of cloudwater at a clean air site in central Scandinavia," *Tellus. Series B: Chemical and Physical Meteorology*, vol. 38, pp. 190–196, 1986.

[22] D. J. Jacob, J. M. Waldman, M. Haghi, M. R. Hoffmann, and R. C. Flagan, "Instrument to collect fogwater for chemical analysis," *Review of Scientific Instruments*, vol. 56, no. 6, pp. 1291–1293, 1985.

[23] C. Leuenberger, J. Czuczwa, E. Heyerdahl, and W. Giger, "Aliphatic and polycyclic aromatic hydrocarbons in urban rain, snow and fog," *Atmospheric Environment*, vol. 22, no. 4, pp. 695–705, 1988.

[24] W.-M. G. Lee and J.-L. Yeh, "Sampling and analysis of atmospheric fog in the suburban area of a severely polluted city," *Journal of Aerosol Science*, vol. 26, no. suppl 1, pp. S383–S384, 1995.

[25] M. Sasakawa and M. Uematsu, "Chemical composition of aerosol, sea fog, and rainwater in the marine boundary layer of the northwestern North Pacific and its marginal seas," *Journal of Geophysical Research. D: Atmospheres*, vol. 107, no. 24, art. 4783, 2002.

[26] M. Sasakawa, A. Ooki, and M. Uematsu, "Aerosol size distribution during sea fog and its scavenge process of chemical substances over the northwestern North Pacific," *Journal of Geophysical Research. D: Atmospheres*, vol. 108, no. 3, art. 4120, 2003.

[27] C. J. Schomburg, D. E. Glotfelty, and J. N. Seiber, "Pesticide occurrence and distribution in fog collected near Monterey, California," *Environmental Science & Technology*, vol. 25, no. 1, pp. 155–160, 1991.

[28] S. M. Chernyak, C. P. Rice, and L. L. McConnell, "Evidence of currently-used pesticides in air, ice, fog, seawater and surface microlayer in the Bering and Chukchi seas," *Marine Pollution Bulletin*, vol. 32, no. 5, pp. 410–419, 1996.

[29] A. T. Vermeulen, G. P. Wyers, F. G. Römer, N. F. M. Van Leeuwen, G. P. J. Draaijers, and J. W. Erisman, "Fog deposition on a coniferous forest in The Netherlands," *Atmospheric Environment*, vol. 31, no. 3, pp. 375–386, 1997.

[30] V. Elias, M. Tesar, and J. Buchtele, "Occult precipitation: sampling, chemical analysis and process modelling in the Sumava Mts., (Czech Republic) and in the Taunus Mts. (Germany)," *Journal of Hydrology*, vol. 166, no. 3, pp. 409–420, 1995.

[31] K. D. Kimball, R. Jagels, G. A. Gordon, K. C. Weathers, and J. Carlisle, "Differences between New England coastal fog and mountain cloud water chemistry," *Water, Air, & Soil Pollution*, vol. 39, no. 3-4, pp. 383–393, 1988.

[32] J. W. Munger, J. L. Collett Jr., B. C. Daube Jr., and M. R. Hoffmann, "Fogwater chemistry at Riverside, California," *Atmospheric Environment. Part B. Urban Atmosphere*, vol. 24, no. 2, pp. 185–205, 1990.

[33] M. Igawa, J. W. Munger, and M. R. Hoffmann, "Analysis of aldehydes in cloud- and fogwater samples by HPLC with a postcolumn reaction detector," *Environmental Science & Technology*, vol. 23, no. 5, pp. 556–561, 1989.

[34] J. W. Munger, J. L. Collett Jr., B. C. Daube Jr., and M. R. Hoffmann, "Chemical composition of coastal stratus clouds: dependence on droplet size and distance from the coast," *Atmospheric Environment*, vol. 23, no. 10, pp. 2305–2320, 1989.

[35] P. Herckes, T. Lee, L. Trenary, G. Kang, H. Chang, and J. L. Collett Jr., "Organic matter in central California radiation fogs," *Environmental Science & Technology*, vol. 36, no. 22, pp. 4777–4782, 2002.

[36] J. L. Collett Jr., B. C. Daube Jr., and M. R. Hoffmann, "The chemical composition of intercepted cloudwater in the Sierra Nevada," *Atmospheric Environment. Part A. General Topics*, vol. 24, no. 4, pp. 959–972, 1990.

[37] K. F. Moore, D. Eli Sherman, J. E. Reilly, and J. L. Collett Jr., "Development of a multi-stage cloud water collector. Part 1: Design and field performance evaluation," *Atmospheric Environment*, vol. 36, no. 1, pp. 31–44, 2002.

[38] K. F. Moore, D. Eli Sherman, J. E. Reilly, and J. L. Collett Jr., "Drop size-dependent chemical composition in clouds and fogs. Part I. Observations," *Atmospheric Environment*, vol. 38, no. 10, pp. 1389–1402, 2004.

[39] D. J. Jacob, R. F. T. Wang, and R. C. Flagan, "Fogwater collector design and characterization," *Environmental Science & Technology*, vol. 18, no. 11, pp. 827–833, 1984.

[40] C. A. Johnson, L. Sigg, and J. Zobrist, "Case studies on the chemical composition of fogwater: the influence of local gaseous emissions," *Atmospheric Environment*, vol. 21, no. 11, pp. 2365–2374, 1987.

[41] D. J. Jacob, J. M. Waldman, J. W. Munger, and M. R. Hoffmann, "Chemical composition of fogwater collected along the California coast," *Environmental Science & Technology*, vol. 19, no. 8, pp. 730–736, 1985.

[42] V. A. Mohnen, V. P. Aneja, B. H. Bailey, et al., "An assessment of atmospheric exposure and deposition to high elevation forests in the Eastern United States," Tech. Rep. EPA/600/3-90/058, U.S. Environmental Protection Agency (EPA), Office of Research and Development, Washington, DC, USA, 1990.

[43] R. J. Vong, B. H. Bailey, M. J. Markus, and V. A. Mohnen, "Factors governing cloud water composition in the Appalachian mountains," *Tellus. Series B: Chemical and Physical Meteorology*, vol. 42, no. 5, pp. 435–453, 1990.

[44] A. R. McFarland and C. A. Ortiz, "Characterization of the Mesh Impactor Fog Sampler," Report to Southern California Edison (Research & Development), Texas Engineering Experiment Station Project 325251107, May 1984.

[45] T. P. DeFelice and V. K. Saxena, "Mechanisms for the operation of three cloudwater collectors: comparison of mountaintop results," *Atmospheric Research*, vol. 25, no. 4, pp. 277–292, 1990.

[46] B. Tenberken and K. Bächmann, "Sampling and analysis of single cloud and fog drops," *Atmospheric Environment*, vol. 32, no. 10, pp. 1757–1763, 1998.

[47] S. Fuzzi, G. Cesari, F. Evangelisti, M. C. Facchini, and G. Orsi, "An automatic station for fog water collection," *Atmospheric Environment. Part A. General Topics*, vol. 24, no. 10, pp. 2609–2614, 1990.

[48] S. Fuzzi, M. C. Facchini, G. Orsi, et al., "The NEVALPA project: A regional network for fog chemical climatology over the PO Valley basin," *Atmospheric Environment*, vol. 30, no. 2, pp. 201–213, 1996.

[49] S. Fuzzi, G. Orsi, G. Bonforte, B. Zardini, and P. L. Franchini, "An automated fog water collector suitable for deposition networks: design, operation and field tests," *Water, Air, & Soil Pollution*, vol. 93, no. 1–4, pp. 383–394, 1997.

[50] M. del Monte and P. Rossi, "Fog and gypsum crystals on building materials," *Atmospheric Environment*, vol. 31, no. 11, pp. 1637–1646, 1997.

[51] A. Jiries, "Chemical composition of dew in Amman, Jordan," *Atmospheric Research*, vol. 57, no. 4, pp. 261–268, 2001.

[52] H. Hutorowicz, "Pomiary rosy w kotowie w latach 1956–1960," *Ekologia Polska. Seria A*, vol. 10, p. 255, 1962.

[53] H. Hutorowicz, "Metody pomiaru rosy," *Ekologia Polska. Seria B*, vol. 9, p. 53, 1963.
[54] H. Hutorowicz, "Nowy typ rosografu," *Zeszyty Naukowe WSR w Olsztynie*, vol. 1, p. 154, 1956.
[55] X.-Y. Li, "Effects of gravel and sand mulches on dew deposition in the semiarid region of China," *Journal of Hydrology*, vol. 260, no. 1, pp. 151–160, 2002.
[56] G. J. Kidron, "Analysis of dew precipitation in three habitats within a small arid drainage basin, Negev Highlands, Israel," *Atmospheric Research*, vol. 55, no. 3-4, pp. 257–270, 2000.
[57] M. Muselli, D. Beysens, J. Marcillat, I. Milimouk, T. Nilsson, and A. Louche, "Dew water collector for potable water in Ajaccio (Corsica Island, France)," *Atmospheric Research*, vol. 64, no. 1–4, pp. 297–312, 2002.
[58] N. Takenaka, H. Soda, K. Sato, et al., "Difference in amounts and composition of dew from different types of dew collectors," *Water, Air, & Soil Pollution*, vol. 147, no. 1–4, pp. 51–60, 2003.
[59] E. Scheller, "Amino acids in dew—origin and seasonal variation," *Atmospheric Environment*, vol. 35, no. 12, pp. 2179–2192, 2001.
[60] R. C. Ferrier, A. Jenkins, and D. A. Elston, "The composition of rime ice as an indicator of the quality of winter deposition," *Environmental Pollution*, vol. 87, no. 3, pp. 259–266, 1995.
[61] N. Berg, P. Dunn, and M. Fenn, "Spatial and temporal variability of rime ice and snow chemistry at five sites in California," *Atmospheric Environment. Part A. General Topics*, vol. 25, no. 5-6, pp. 915–926, 1991.
[62] L. C. Duncan, "Chemistry of rime and snow collected at a site in the central Washington Cascades," *Environmental Science & Technology*, vol. 26, no. 1, pp. 61–66, 1992.
[63] J. R. Foster, R. A. Pribush, and B. H. Carter, "The chemistry of dews and frosts in indianapolis, Indiana," *Atmospheric Environment. Part A. General Topics*, vol. 24, no. 8, pp. 2229–2236, 1990.
[64] M. Chiwa, N. Oshiro, T. Miyake, et al., "Dry deposition washoff and dew on the surfaces of pine foliage on the urban- and mountain-facing sides of Mt. Gokurakuji, western Japan," *Atmospheric Environment*, vol. 37, no. 3, pp. 327–337, 2003.
[65] J. P. Lacaux, J. Loemba-Ndembi, B. Lefeivre, B. Cros, and R. Delmas, "Biogenic emissions and biomass burning influences on the chemistry of the fogwater and stratiform precipitations in the African equatorial forest," *Atmospheric Environment. Part A. General Topics*, vol. 26, no. 4, pp. 541–551, 1992.
[66] D. Grosjean and B. Wright, "Carbonyls in urban fog, ice fog, cloudwater and rainwater," *Atmospheric Environment*, vol. 17, no. 10, pp. 2093–2096, 1983.
[67] O. Klemm, R. W. Talbot, and K. I. Klemm, "Sulfur dioxide in coastal New England fog," *Atmospheric Environment. Part A. General Topics*, vol. 26, no. 11, pp. 2063–2075, 1992.
[68] O. Klemm, A. S. Bachmeier, R. W. Talbot, and K. I. Klemm, "Fog chemistry at the New England Coast: Influence of air mass history," *Atmospheric Environment*, vol. 28, no. 6, pp. 1181–1188, 1994.
[69] J. L. Collett Jr., K. J. Hoag, D. Eli Sherman, A. Bator, and L. W. Richards, "Spatial and temporal variations in San Joaquin Valley fog chemistry," *Atmospheric Environment*, vol. 33, no. 1, pp. 129–140, 1999.
[70] C. Anastasio and K. G. McGregor, "Chemistry of fog waters in California's Central Valley: 1. In situ photoformation of hydroxyl radical and singlet molecular oxygen," *Atmospheric Environment*, vol. 35, no. 6, pp. 1079–1089, 2001.
[71] T. Wrzesinsky and O. Klemm, "Summertime fog chemistry at a mountainous site in central Europe," *Atmospheric Environment*, vol. 34, no. 9, pp. 1487–1496, 2000.
[72] P. Herckes, M. P. Hannigan, L. Trenary, T. Lee, and J. L. Collett Jr., "Organic compounds in radiation fogs in Davis (California)," *Atmospheric Research*, vol. 64, no. 1–4, pp. 99–108, 2002.
[73] J. L. Collett Jr., B. C. Daube Jr., D. Gunz, and M. R. Hoffmann, "Intensive studies of Sierra Nevada cloudwater chemistry and its relationship to precursor aerosol and gas concentrations," *Atmospheric Environment. Part A. General Topics*, vol. 24, no. 7, pp. 1741–1757, 1990.
[74] R. S. Schemenauer, C. M. Banic, and N. Urquizo, "High elevation fog and precipitation chemistry in Southern Quebec, Canada," *Atmospheric Environment*, vol. 29, no. 17, pp. 2235–2252, 1995.
[75] R. S. Schemenauer and P. Cereceda, "The quality of fog water collected for domestic and agricultural use in Chile," *Journal of Applied Meteorology*, vol. 31, no. 3, pp. 275–290, 1992.
[76] F. D. Eckardt and R. S. Schemenauer, "Fog water chemistry in the Namib Desert, Namibia," *Atmospheric Environment*, vol. 32, no. 14-15, pp. 2595–2599, 1998.
[77] R. S. Schemenauer and P. Cereceda, "Monsoon cloudwater chemistry on the Arabian Peninsula," *Atmospheric Environment. Part A. General Topics*, vol. 26, no. 9, pp. 1583–1587, 1992.
[78] M. A. Rubio, E. Lissi, and G. Villena, "Nitrite in rain and dew in Santiago city, Chile. Its possible impact on the early morning start of the photochemical smog," *Atmospheric Environment*, vol. 36, no. 2, pp. 293–297, 2002.
[79] H. Puxbaum and W. Tscherwenka, "Relationships of major ions in snow fall and rime at sonnblick observatory (SBO, 3106 m) and implications for scavenging processes in mixed clouds," *Atmospheric Environment*, vol. 32, no. 23, pp. 4011–4020, 1998.
[80] A. J. Dore, M. Sobik, and K. Migała, "Patterns of precipitation and pollutant deposition in the western Sudete mountains, Poland," *Atmospheric Environment*, vol. 33, no. 20, pp. 3301–3312, 1999.
[81] K. S. Bridges, T. D. Jickells, T. D. Davies, Z. Zeman, and I. Hunova, "Aerosol, precipitation and cloud water chemistry observations on the Czech Krusne Hory plateau adjacent to a heavily industrialised valley," *Atmospheric Environment*, vol. 36, no. 2, pp. 353–360, 2002.
[82] K. Migała, J. Liebersbach, and M. Sobik, "Rime in the Giant Mts. (The Sudetes, Poland)," *Atmospheric Research*, vol. 64, no. 1–4, pp. 63–73, 2002.
[83] A. Woś, *Meteorologia dla geografów*, Wydawnictwo Naukowe PWN, Warszawa, Poland, 2000.
[84] K. Różdżyński, *Miernictwo Meteorologiczne*, vol. 2, Instytut Meteorologii i Gospodarki Wodnej, Warszawa, Poland, 1996.
[85] P. McComber, J. Druez, and J. Laflamme, "A comparison of selected models for estimating cable icing," *Atmospheric Research*, vol. 36, no. 3-4, pp. 207–220, 1995.
[86] J. Fisak, J. Chum, J. Vojta, and M. Tesar, "Instrument for measurement of the amount of the solid precipitation deposit—ice meter," *Journal of Hydrology and Hydromechanics*, vol. 49, no. 3-4, pp. 187–199, 2001.

